# Measuring body composition in overweight individuals by dual energy x-ray absorptiometry

**DOI:** 10.1186/1471-2342-5-1

**Published:** 2005-03-04

**Authors:** Rhonda A Brownbill, Jasminka Z Ilich

**Affiliations:** 1 School of Allied Health, University of Connecticut, Storrs, CT 06269, USA

## Abstract

**Background:**

Dual energy x-ray absorptiometry (DXA) is widely used for body composition measurements in normal-weight and overweight/obese individuals. The limitations of bone densitometers have been frequently addressed. However, the possible errors in assessing body composition in overweight individuals due to incorrect positioning or limitations of DXA to accurately assess both bone mineral density and body composition in obese individuals have not received much attention and are the focus of this report.

**Discussion:**

We discuss proper ways of measuring overweight individuals and point to some studies where that might not have been the case. It appears that currently, the most prudent approach to assess body composition of large individuals who cannot fit under the scanning area would be to estimate regional fat, namely the regions of thigh and/or abdomen. Additionally, using two-half body scans, although time consuming, may provide a relatively accurate measurement of total body fat, however, more studies using this technique are needed to validate it.

**Summary:**

Researchers using bone densitometers for body composition measurements need to have an understanding of its limitations in overweight individuals and address them appropriately when interpreting their results. Studies on accuracy and precision in measurements of both bone and soft tissue composition in overweight individuals using available densitometers are needed.

## Background

Dual energy x-ray absorptiometry (DXA) is widely used by clinicians and researchers for evaluation of bone status and soft tissue composition. While the principles of DXA technology could be found elsewhere [1-3] and are not the focus of this report, we address them briefly for better understanding of the discussion to follow. The underlying principle of DXA is its ability to quantify the attenuated radiation after its passage through bone and soft tissue using either K-edge filters or pulsed power sources to the x-ray tube. Subsequently, the differential attenuation of the two energies is utilized to quantify bone, lean, and/or fat tissue. The earlier DXA series are based on pencil-beam absorptiometry, where a highly collimated x-ray beam and a detector move along the rectilinear scan path. The new series employ fan-beam absorptiometry in which data are acquired either simultaneously along the entire scan line, or as rectilinear scanning with a narrow fan-beam, both resulting in a faster scanning time [1]. The fan beam densitometers have the advantage of improved geometrical resolution, but the disadvantage of errors induced by magnification effects. Within the fan beam instruments, the *true *fan beam densitometers have greater accuracy and precision, shorter scan time, and wider scan field than *limited-angle *fan beam densitometers which have inherent overlap in acquisition, smaller number of detectors, and poorer image quality [3].

The three major commercial manufacturers of bone densitometers are GE Medical Systems Inc. (former Lunar), Madison, WI; Hologic Inc., Waltham, MA; and CooperSurgical (former Norland Medical Systems, Inc.), Trumbull, CT. Although each of these companies employs a subtly different technology, our further discussion does not address the particulars of each technology and/or manufacturer. Our focus is the positioning of the overweight patients when obtaining densitometry scans and subsequent analyses of these scans, overlooked in many studies. However, for more information, the main physical characteristics of the most commonly used manufacturer/instruments are presented in Table 1.

DXA is considered one of the most precise technologies in clinical medicine when the measurement of bone mineral density (BMD) is considered, with the typical coefficients of variation between 1–2% [4]. Nevertheless, there are some limitations in BMD assessment as well. Results of in vitro and in vivo studies indicate different manufacturers, models, software versions and modes of analysis of densitometers can lead to variations in the assessed BMD and bone mineral content (BMC) in the same individuals [5,6]. Laskey et al (2004) found that the GE Lunar Prodigy gave significantly higher BMD, BMC and t-scores compared to the GE Lunar MD in 10 volunteers [6]. They also found that an increase in tissue depth (as in overweight individuals) caused an increase in the measured BMC and BMD for the MD model but not the Prodigy model, even when using the appropriate and same scan modes [6]. The prudent way to overcome these flaws would be to use the same instrument and software version throughout a single longitudinal study.

The accuracy of DXA instruments for measurement of soft tissue is also questioned due to various methodological limitations. Some of the limitations are addressed in a recent review [7] and are generally attributed to the hardware (fan- or pencil-beam) or software versions [8]. DXA instruments from different manufactures are shown to give considerably different soft tissue assessments of the same individual [9]. Lunar and Hologic are shown to give major differences in measurements of total body and regional body fat in HIV patients (2.4–13.4% higher values for Hologic) and in body fat distribution [9]. Additionally, individuals' hydration levels may affect calculations for soft tissue [7] whereas tissue thickness may affect beam magnification, especially if the proper scan mode is not chosen and in cases involving changes in subject's weight [10]. Also, estimates for soft tissue in regions directly adjacent to the large bony areas such as the trunk, arms and head, may result in decreasing precision. During the total body scans (to obtain body composition analysis) a larger pixel size is utilized and pixels that include smaller portions of bone may be counted as lean tissue [10]. Despite the above flaws, DXA can still be used for fairly accurate assessment of soft tissue composition or its change [7], particularly for groups and large-scale epidemiological studies, provided that its limitations are considered and adequately accounted for. However, it has to be noted that DXA technology is not approved by Food and Drug Administration for the individual assessment of body composition.

Currently, the most accurate method for measuring body composition is considered to be the four-compartment (4C) model in which fat free body tissue is divided into its constituent parts, namely water, protein and mineral. The 4C model then incorporates independent measurements of mineral, total body water and body density to derive body fat. The 4C model (though not a true gold standard) is often used as a criterion method to compare the accuracy of other methods for assessing body fat. This method however, is costly and time consuming and therefore not generally used in clinical settings. DXA (a two-compartment method) does not measure body water, which limits its accuracy in body composition assessment. However, since DXA offers quick and easy body fat assessment and is considered superior to many other methods, it is often used in clinical settings. Gately et al. [11] compared various body composition methods for assessing body fat in overweight and obese children. They found air-displacement plethysmography and DXA to be the most promising methods for body fat assessment in a clinical setting [11]. A study in non-obese women found DXA to be superior to waist circumference and waist-to-hip ratio in predicting intra abdominal fat [12].

The use of DXA for assessment of body composition in overweight/obese individuals increased recently due to numerous weight reduction studies. While all of the above limitations of bone densitometers have been frequently addressed, the limitations of assessing body composition in overweight individuals due to incorrect positioning and subsequent failures to properly analyze the obtained scans have not received any attention and are the focus of this report. We discuss proper ways of measuring overweight individuals and assessing their soft tissue and point to some studies where that may not have been the case.

## Discussion

### Use of bone densitometers in weight loss studies

In weight loss studies where DXA is used to evaluate lean and fat tissue, overweight/obese individuals range widely in body weight and size [8,13-20]. However, the maximum size of a DXA scanning table is limited to about 193–197 cm length and 58–65 cm width, with weight limitations from 114–159 kg depending on the manufacturer and model, Table 1. In order to fit an overweight individual within the scanning area, rice bags and straps are used to press the limbs as close to the body as possible [2]. Despite these measures, some large individuals cannot fit within the global region of the scan area. Additionally, in some cases, the space between the scanning table and the detector is not large enough to accommodate individuals with a larger chest girth, making their measurements difficult or impossible.

**Figure 1:**
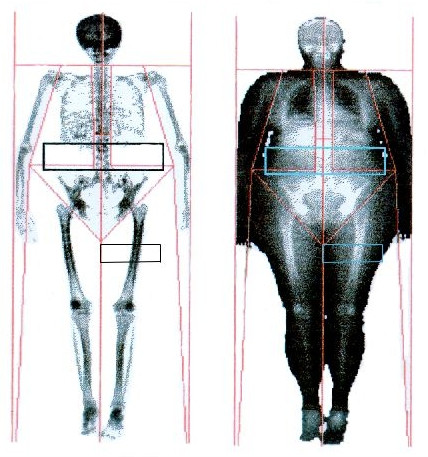
Total body scan, with bones only (left) and with soft tissue (right), of a 104 kg woman (BMI = 34.1 kg/m^2^). Portions of the arms fell out of the scan area and there is overlapping tissue in the chest, arm, and hip regions. The abdominal and thigh regions of interest can be defined manually, while others are determined by computer software, although they can be manually changed as well.

**Figure 2:**
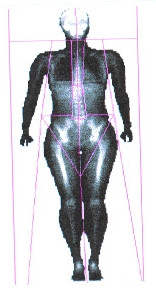
Total body scan of a 59 kg woman (BMI = 22.6 kg/m^2^) showing proper positioning on the scan table and no overlapping regions.

While some authors do address these limitations when reporting their data [14], some do not describe or vaguely describe DXA assessment [13,15,17,19] or are unclear regarding precision of their instruments in overweight individuals [15-18,20]. In our own preliminary studies with overweight women using a Lunar pencil-beam densitometer, the coefficients of variation (CV) for different skeletal sites ranged from 0.6–1.8% [21], but those for the soft tissue were higher reaching 8.2% for fat tissue in the arms (not published). The high %CVs (range 3.1–4.3%) for fat tissue (even in normal-weight individuals) were reported by others using pencil-beam instruments [22]. Figure 1 shows a total body scan of a 104 kg woman where portions of the arms fell out of the scan area, and therefore, could not be included in the analysis of the total body soft tissue. Furthermore, since her limbs were pressed against the sides of her body, overlap of tissue occurred in the chest, arm and hip regions, resulting in inaccurate regional soft tissue analysis (namely, trunk, legs and arms). Figure 2 presents the proper positioning and analysis of total body composition in a 59 kg woman. It is obvious that the inclusion of subjects who do not fit in the global scan area might lead to questionable accuracy of both total and regional soft tissue estimates.

### Total body and regional soft tissue assessment

When total body soft tissue assessment is the goal, it is necessary to include all parts of the body in the scanning area. In overweight subjects, overlapping of body parts may affect the total results due to increased thickness in overlapping regions. Another source of error is the head, where tissue type cannot be distinguished. Specifically, the brain tissue cannot be measured by DXA due to the surrounding skull – it has to be assumed. Therefore, the assessment of soft tissue in this region is subject to large error and it is suggested the head be excluded from total body soft tissue analysis [10]. In regional assessment, DXA utilizes the placement of standard cut-lines to assess the arms, legs and trunk (chest, abdomen, pelvis), Figure 1 and 2. Each regional estimate may be subject to error in overweight individuals (in normal-weight ones too) if overlapping of regions occurred. Wang et al. [13] measured total and regional body fat with DXA in women (mean ± SD weight, 96 ± 11 kg) before and after weight loss. Since the positioning of the subjects was not described, it is not known whether all subjects fit within the scan area and whether tissue overlap occurred. Similar uncertainty exists in other studies [18,19].

In the newest study by Sun et al. [20] researchers compared the assessment of total body fat by multi-frequency bioelectrical impedance with DXA measurements as the "gold standard". The subjects in the study ranged in weight from 45 to 157 kg, with body mass index (BMI) ranging from 17 to 55 kg/m^2^, indicating some were severely obese. However, authors did not address the positioning or fitting of the obese subjects on the scanning table, therefore it could only be speculated about the adequacy of these measurements/analyses.

Researchers have found estimates of abdominal fat tissue by DXA to be similar to computed tomography (CT) and MRI-derived measurements in normal and overweight individuals of wide age range, indicating DXA can accurately estimate abdominal fat [24-26]. The abdominal region is not a routinely defined region by DXA software and therefore, must be manually determined (see Figure 1), which can differ among research sites. Park et al. [24] compared abdominal adipose tissue measured by MRI and DXA in non-obese men. They defined DXA regions of interest in two different ways (between the second-lumbar vertebra and the fourth-lumbar vertebra, or iliac crest) and found both of these regions comparable with MRI total abdominal adiposity and with MRI-derived narrow abdominal slices. Bertin et al. [26] reported DXA yielded accurate measurements of abdominal adipose tissue compared with CT in overweight/obese individuals weighing 66–134 kg. They manually defined DXA abdominal region to range from the acromion to the iliac crest, a slightly different placement than the ones described above, and compared it to a 10 mm region at the fourth-lumbar vertebrae measured by CT. It is important to note that the abdominal regions of interest could be subject to potential error if the upper limbs are positioned in too close contact with the trunk, causing the overlap of the regions.

Researchers have also found estimates of different regions of leg soft tissue extracted from the total DXA scans to be reliable in elderly subjects of wide weight range [8,26,27]. Similarly, fat tissue of the thigh determined by DXA was comparable to CT derived measurements in normal and overweight individuals. Tylavsky et al. [8] compared CT derived measurements of lean and fat tissue with DXA measurements of a manually defined sub-region of the mid-thigh (one-half the distance between the knee joint and the top of the femur, see Figure 1). They indicated a good assessment of soft tissue change by DXA in that region. It therefore appears that with large individuals, DXA should be used for assessing body composition of defined regions such as the mid-thigh or abdominal rather than the total body.

### Half-body DXA scans for the assessment of soft tissue

Tataranni and Ravussin [28] suggest measuring soft tissue of obese individuals by scanning only one side of the body. They found total body composition results from right and left sides only differed minimally in both overweight and normal-weight individuals. The half-body scan can be performed by placing the central line of the scanning area through the midpoint of the left or right collar bone for each half-body scan. During analysis of the half-body scans, the central line is then repositioned on each half scan, and the side of the body that was not completely included in the scan area is deleted. The authors found that small errors in estimates of soft tissue can occur from imperfect positioning of the central line by the operator or by true anatomical differences between the left and right sides of the body. To minimize these errors, they suggest fat tissue be determined by multiplying percent body fat from the half-body scan by body weight, and lean tissue be determined as the difference between body weight and estimated fat tissue. Another possibility for improving accuracy of soft tissue assessment would be measuring both halves of the body, and then adding them up. However, more research on the above methods is necessary in order to make recommendations

### Total body bone mineral assessment in overweight individuals

Similarly to problems with soft tissue assessment, there are problems with bone mass assessment when DXA is used in overweight individuals. When an individual does not fit within the scan region, there is subsequent loss of soft and bone tissue. Additionally, some anomalies in bone mass measurement during weight loss studies using different instruments were reported earlier [29]. Tothill et al. [29], re-analyzed published results of changes in total body bone mineral during weight change. The authors found weight change leads to considerable anomalies in measuring changes in bone mineral in all three brands of DXA machines (Hologic, Lunar and Norland), with the most serious ones occurring with Hologic [29]. These inaccuracies were suspected to be due to the use of different software modes (enhanced vs. standard) and the different assumptions manufactures make regarding fat distribution [29]. Phantom studies using Lunar and Hologic fan beam scanners showed bone mass measurements were not compromised by magnification effects, however, the height of bone and changes in body weight simulated with lard did affect the accuracy of BMD and BMC measurements [30]. Tothill and Hannan [30] compared Lunar and Hologic DXA fan bean scanners for measuring total body bone and soft tissue. Phantom measurements revealed that both fan beam instruments were subject to minor magnification effects, and measurements of BMD and BMC were both dependent on the height of a bone [30].

## Summary

Current bone densitometers are limited to a scanning area that cannot accommodate some overweight/obese individuals. Newer fan-beam densitometers have a wider scan field [3] or can accommodate individuals up to 159 kg, Table 1, making them a better option for body composition assessment. Unless researchers are using some of the newer densitometers (with a scan table large enough to accommodate larger body sizes) they may need to rely on estimates of regional fat, namely the thigh or abdominal region when assessing the body composition of many overweight subjects. Using one or two half-body scans may provide a relatively accurate measurement of total body fat, however, more studies using this technique are needed. The results of some published studies in overweight/obese individuals need to be interpreted with caution, since they may have included subjects who could not properly fit within the scan area. Researchers using bone densitometers for body composition measurements need to have an understanding of its limitations in overweight individuals and appropriately address the stated concerns when interpreting their results. Authors also need to provide details of their DXA instrument including the manufacturer, the software version and the analysis mode used for body composition assessment when reporting their results. Studies on accuracy and precision in measurements of both bone and soft tissue composition in overweight individuals, using available densitometers, are warranted and needed.

## Competing interests

The author(s) declare that they have no competing interests.

## Authors' contributions

RAB wrote article drafts and evaluated the cited literature. JZI conceptualized the idea and the design of the article, revised the manuscript and completed the final version. Both authors read and approved the final manuscript.

## Pre-publication history

The pre-publication history for this paper can be accessed here:



## Figures and Tables

**Table 1:** Weight limits and table dimensions of full-size densitometers of various manufactures and models

Manufacturer/ Densitometer	Weight Limit kg (lb)	Table Dimensions cm
GE Lunar Prodigy Advance	159 (350)	197.5 × 60
GE Lunar Prodigy	136 (300)	197.5 × 60
GE Lunar DPX-MD	136 (300)	196.8 × 57.6
Hologic QDR series	136 (300)	195.6 × 65- 67
Hologic Discovery Series	159 (350)	195.6 × 67
Norland XR-46	114 (250)	193 × 64
Norland XR-36	114 (250)	193 × 64
